# Dataset on anti-human insulin-degrading enzyme activities of cyclic tetra peptides: Insight from insilico approach

**DOI:** 10.1016/j.dib.2024.110724

**Published:** 2024-07-14

**Authors:** Abel K. Oyebamiji, Faith Eniola Olujinmi, Halleluyah O. Aworinde, David G. Oke, Sunday Adewale Akintelu, Emmanuel T. Akintayo, C.O. Akintayo, Jonathan O. Babalola

**Affiliations:** a Industrial Chemistry Programme, Bowen University, Iwo, Osun State, Nigeria; b College of Computing and Communication Studies, Bowen University, Iwo, Nigeria; c Department of Pure and Applied Chemistry, Ladoke Akintola University of Technology, Ogbomoso, Oyo State, Nigeria; d Department of Chemistry, Ekiti State University, Ado-Ekiti, Nigeria; e Department of Chemistry, Federal University, Oye-Ekiti, Ekiti State, Nigeria; f Department of Chemistry, University of Ibadan, Ibadan, Nigeria; g Good Health and Wellbeing Research Clusters (SDG 03), Bowen University, PMB 284 Iwo, Nigeria

**Keywords:** Functionalized, Insulin, Peptides, Enzymes, Inhibitors, DFT, MD

## Abstract

In this work, the biochemical activities of seven cyclic peptides were investigated using the *insilico* approach. The materials used in this work were Spartan 14 for quantum chemical analysis, molecular operating environment software for molecular docking and ADMETSAR 2.0 for pharmacokinetic investigation. The calculated features obtained for each compound were explored and it was observed that the molecules used in this research have potential anti-human insulin-degrading enzyme activities. Also, (3S,6S,9S)-9-((*R*)-1-(benzyloxy)ethyl)-6-methyl-3-(4-methylphenethyl)-1,4,7,10-tetraazacyclododecane-2,5,8,11-tetraone (compound 2) with highest binding affinity (−7.95349026 kcal/mol) possess utmost ability to inhibit human insulin-degrading enzyme (PDB id: 2g56) than other investigated compounds and acarbose (referenced compound). The pharmacokinetic analysis for compound 2 was examined and compared to the predicted report for the referenced compound.

Specification Table

**Table:** 

Subject	Computational Study
Specific subject area	Drug prediction
Type of data	Figure, Chart, Table, ADMET, Graph
Data collection	The examined compounds were optimized in gas, water and ethanol phases to investigate their activities in different solvents. The inhibiting activities of the optimized compounds against human insulin-degrading enzyme (PDB id: 2g56) were calculated via molecular modelling methods (Molecular docking and molecular dynamic simulation) using molecular operating environment (MOE) software. The absorption, distribution, metabolism, excretion and toxicity analysis for the lead compound were examined using admetsar software. All the obtained results were presented and explained.
Data source location	Computational Chemistry Research Laboratory, Department of Chemistry and Industrial Chemistry, BOWEN University, PMB 284, Iwo, Osun State, Nigeria.
Data accessibility	Repository name: Mendeley Data Data identification number: 10.17632/jjvwp35k78.2 Direct URL to data: https://data.mendeley.com/datasets/jjvwp35k78/2 Instructions for accessing these data: The data can be accessed using the above URL
Related research article	*None*

## 1 Value of the Data

• The obtained descriptors from the optimized compounds in different phases (vacuum, water, and ethanol) will expose to scientists the solvent that is suitable for the solubility of the examined compounds.
• The predicted data from the optimized compounds will help scientists to know the level of reactivity of the optimized compounds.
• The calculated binding affinity for the investigated compounds against human insulin-degrading enzyme (PDB id: 2g56) will reveal to researchers the compound that has the best ability to inhibit the examined receptor.
• The predicted binding energy, root mean square deviation (rmsd), root means square fluctuation (rmsf), the radius of gyration (rog) and hydrogen bond calculation will help researchers to confirm compounds with the highest ability to inhibit the human insulin-degrading enzyme (PDB id: 2g56).
• The predicted pharmacokinetics will assist researchers in discerning the probable biochemical activities of individual ligands in the human body system.

## 2 Background

The objectives of this work are:

➢ To examine the effect of solvent on the reactivity of the investigated cyclic peptides.
➢ To observe the biochemical activities of the compounds in the active site of human insulin-degrading enzyme (PDB id: 2g56).
➢ To reveal the level of toxicity of the lead compound.

## 3 Data Description

The structure and IUPAC names of the examined compounds are displayed in Table 1. Table 1 comprises seven compounds and the parent compound was attached to each of the following atom/molecules: Hydrogen, Methylbenzene, Methylbenzoate, Flourobenze, 3,5-dimethylbenzene, Chlorobenzene and Bromobenzene to form individual compound investigated in this work.

**Table 1:** Structure and IUPAC names of the Investigated compounds.

Table 2 revealed the descriptors which described the activities of the examined compounds and the descriptors obtained from the investigated phases were highest occupied molecular orbital energy (EHOMO), lowest unoccupied molecular orbital energy (ELUMO) and energy gap.

**Table 2:** Predicted descriptors from the optimized compounds in Gas Phase, water and Ethanol.

	Gas phase			Water phase			Ethanol phase		
	HOMO	LUMO	EG	HOMO	LUMO	EG	HOMO	LUMO	EG
1	−6.51	−0.81	5.70	−6.65	−0.28	6.37	−6.53	−0.27	6.26
2	−6.33	−0.49	5.84	−6.19	−0.52	5.67	−6.18	−0.13	6.05
3	−6.61	−1.01	5.6	−6.64	−1.22	5.42	−6.38	−1.37	5.01
4	−6.22	−0.54	5.68	−6.29	−0.33	5.96	−6.25	−0.36	5.89
5	−6.26	−0.35	5.91	−6.22	−0.39	5.83	−6.21	−0.36	5.85
6	−6.43	−0.78	5.65	−6.37	−0.42	5.95	−6.42	−0.29	6.13
7	−6.51	−0.51	6.00	−6.40	−0.31	6.09	−6.40	−0.31	6.09

The precise locations for HOMO-LUMO overlay on the compounds under investigation were displayed in Table 3, Table 4, Table 5. The HOMO-LUMO overlay for the compound optimized in vacuum, water and ethanol was represented using solid, mesh and transparent format respectively.

**Table 3:** Predicted HOMO–LUMO overlay for examined cyclic peptide-based compound in Gas phase.

**Table 4:** Predicted HOMO–LUMO overlay for examined cyclic peptide-based compound in Water Phase.

**Table 5:** Predicted HOMO–LUMO overlay for examined cyclic peptide-based compound in Ethanol Phase.

**Fig. 1:**
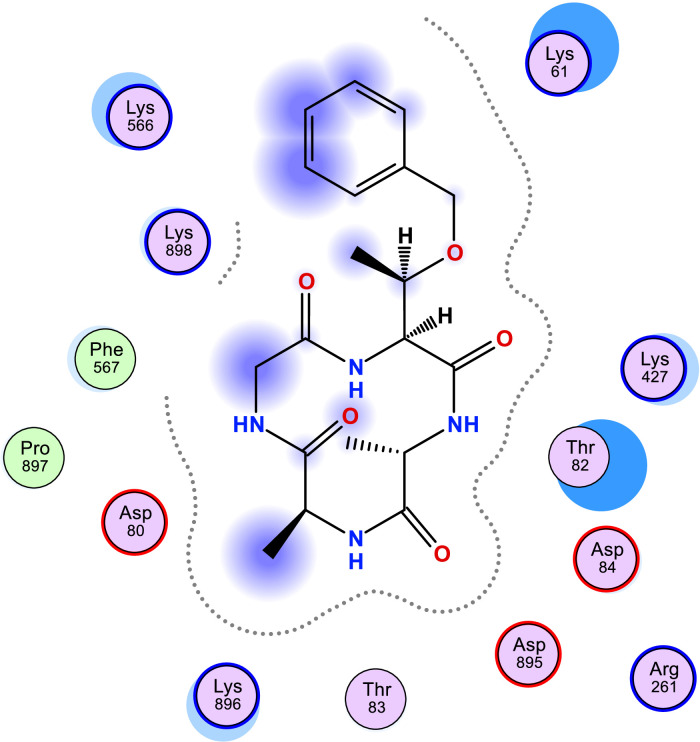
Two Dimensional structure of compound 1 docked with human insulin-degrading enzyme (PDB id: 2g56).

**Fig. 2:**
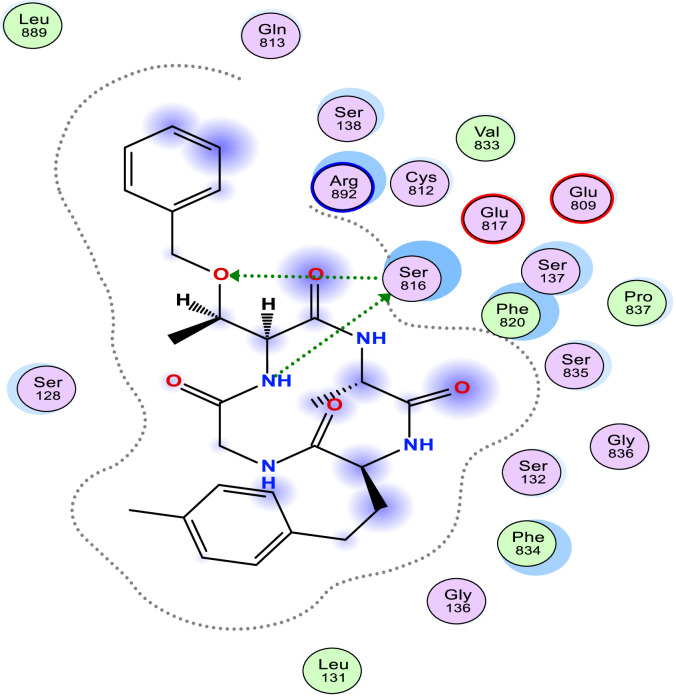
Two Dimensional structure of compound 2 docked with human insulin-degrading enzyme (PDB id: 2g56).

**Fig. 3:**
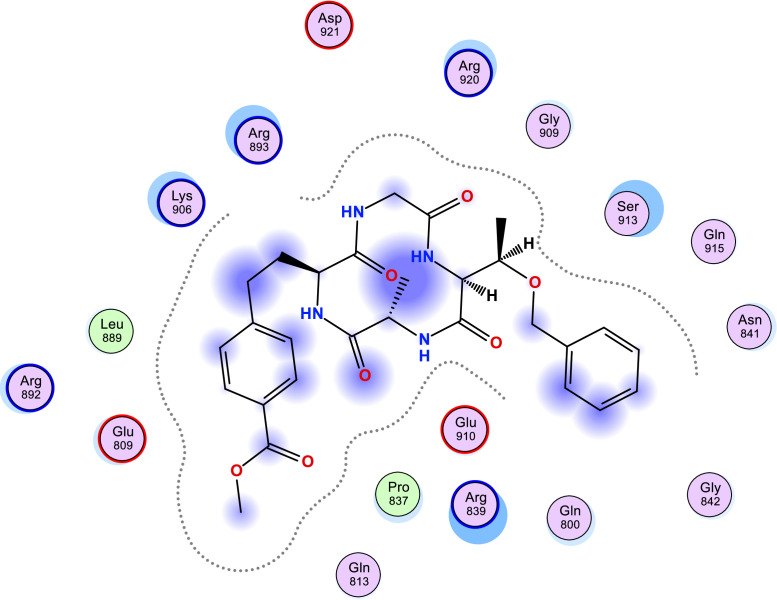
Two Dimensional structure of compound 3 docked with human insulin-degrading enzyme (PDB id: 2g56).

**Fig. 4:**
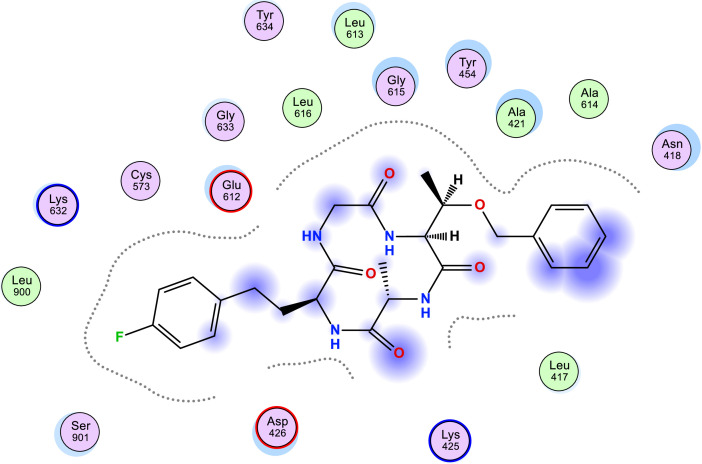
Two Dimensional structure of compound 4 docked with human insulin-degrading enzyme (PDB id: 2g56).

**Fig. 5:**
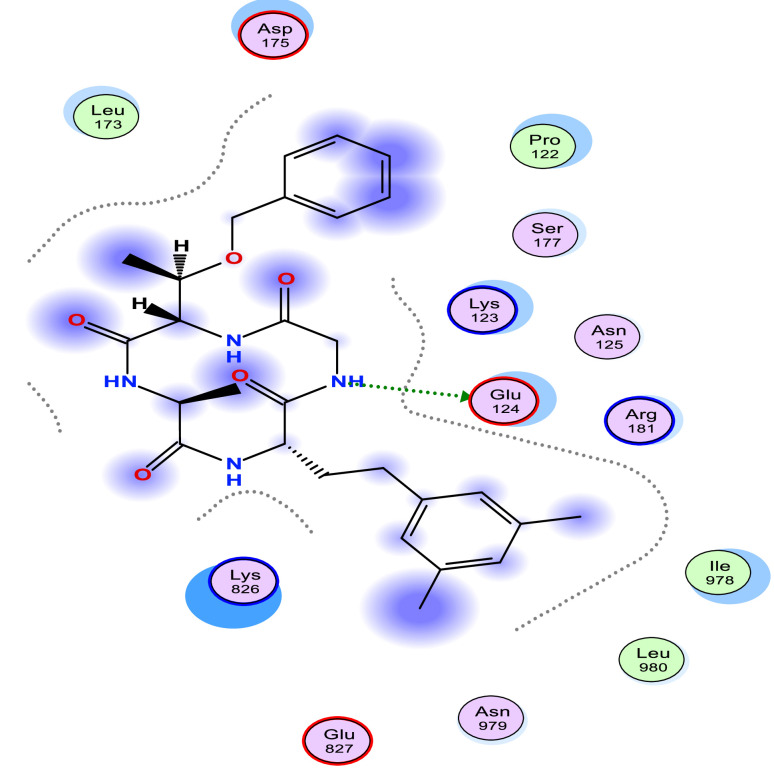
Two Dimensional structure of compound 5 docked with human insulin-degrading enzyme (PDB id: 2g56).

**Fig. 6:**
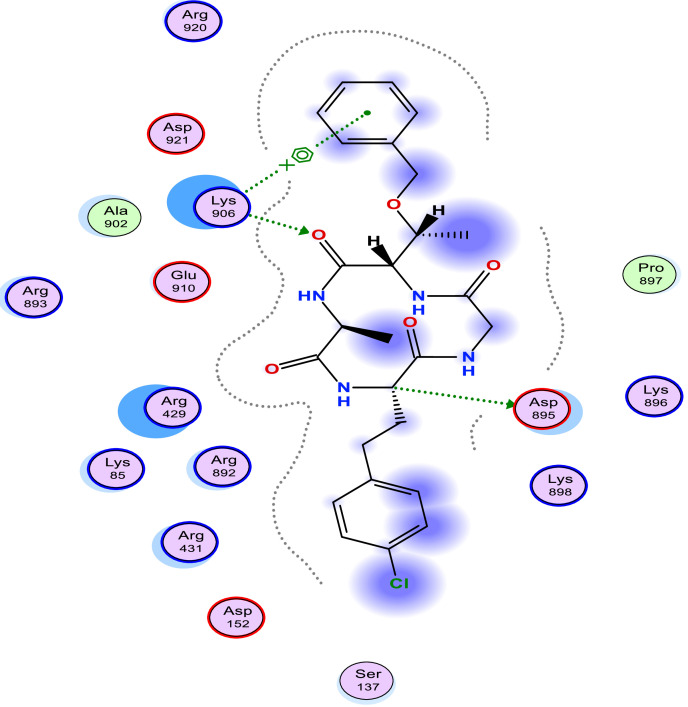
Two Dimensional structure of compound 6 docked with human insulin-degrading enzyme (PDB id: 2g56).

**Fig. 7:**
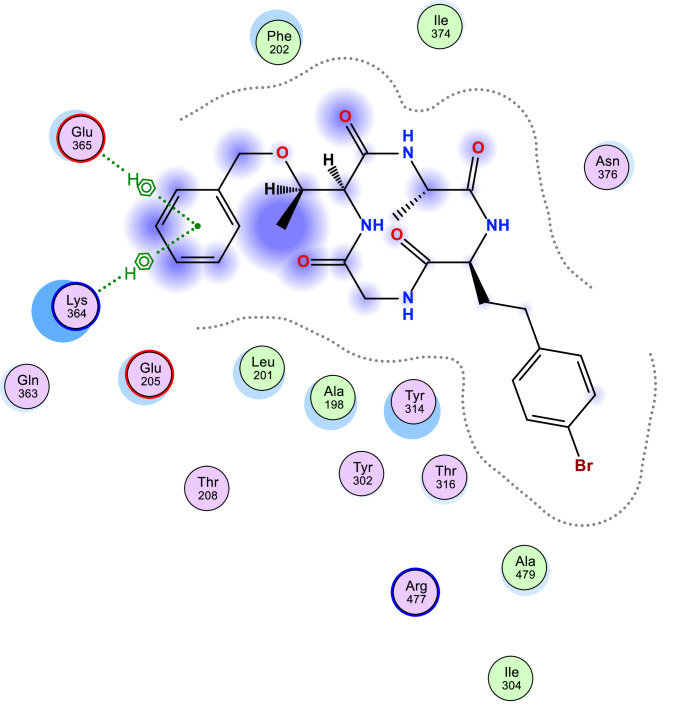
Two Dimensional structure of compound 7 docked with human insulin-degrading enzyme (PDB id: 2g56).

Table 6 shows the calculated scoring for the docked cyclic peptide-based compounds against human insulin-degrading enzyme (PDB id: 2g56). The calculated scoring was −6.60211849 kcal/mol for compound 1, −7.95349026 kcal/mol for compound 2, −7.39044142 kcal/mol for compound 3, −7.03656101 kcal/mol for compound 4, −6.98292017 kcal/mol for compound 5, −7.21901321 kcal/mol for compound 6, −7.06470251 kcal/mol for compound 7 and they were compared with the calculated binding affinity for the reference compound (−7.69367123 kcal/mol). Also, the type of interaction, the amino acid involved in the interaction, the distance and the part of the ligand involved in the interaction were presented in Table 7 and the 2-dimensional structure of the ligand in the active site of the investigated receptor were presented in Fig. 1, Fig. 2, Fig. 3, Fig. 4, Fig. 5, Fig. 6, Fig. 7.

**Table 6:** Calculated binding affinity for Cyclic Peptides based compounds and human insulin-degrading enzyme (PDB id: 2g56).

	Scoring (kcal/mol)
1	−6.60211849
2	−7.95349026
3	−7.39044142
4	−7.03656101
5	−6.98292017
6	−7.21901321
7	−7.06470251
ref	−7.69367123

Ref: Acarbose.

**Table 7:** Nonbonding interaction involved in the docked complexes.

Compounds	Types of Interactions				
1	−				
2	Ligand	Receptor	Interaction	Distance	E (kcal/mol)
	N 3 OG O 52 OG	SER 816 (A) SER 816 (A)	H-donor H-acceptor	2.80 2.87	−2.6 −0.6
3	−				
4	−				
5	Ligand	Receptor	Interaction	Distance	E (kcal/mol)
	N 37 OE2	GLU 124(A)	H-donor	3.26	−1.9
6	Ligand	Receptor	Interaction	Distance	E (kcal/mol)
	C 27 OD2 O 2 NZ 6-ring NZ	ASP 895 (A) LYS 906 (A) LYS 906 (A)	H-donor H-acceptor pi-cation	3.28 2.94 3.69	−1.1 −8.8 −0.8
7	Ligand	Receptor	Interaction	Distance	E (kcal/mol)
	6-ring CA 6-ring N	LYS 364(A) GLU 365(A)	pi-H pi-H	3.96 4.23	−1.1 −1.6
ref	Ligand	Receptor	Interaction	Distance	E (kcal/mol)
	O 10 OE1 O 12 OE2 O 16 OE2 O 11 ND2 O 18 OG1	GLU 817(A) GLU 133(A) GLU 880(A) ASN 821(A) THR 878(A)	H-donor H-donor H-donor H-acceptor H-acceptor	3.19 3.07 3.34 2.93 3.11	−1.3 −1.2 −0.5 −0.9 −0.6

Table 8, Table 9 show the pharmacokinetic analysis of the lead (compound 2) and reference compounds. The analysis was presented in ADMET predicted profile — classification and ADMET predicted profile — regression format. The prediction was displayed under three headings (model, result and probalility) and the predicted model for absorption were Blood–Brain Barrier, Human Intestinal Absorption, Caco-2 Permeability, P-glycoprotein Substrate, P-glycoprotein Inhibitor and Renal Organic Cation Transporter; for distribution was Subcellular localization; metabolism were CYP450 2C9 Substrate, CYP450 2D6 Substrate, CYP450 3A4 Substrate, CYP450 1A2 Inhibitor, CYP450 2C9 Inhibitor, CYP450 2D6 Inhibitor, CYP450 2C19 Inhibitor, CYP450 3A4 Inhibitor and CYP Inhibitory Promiscuity and for toxicity, the Human Ether-a-go-go-Related Gene Inhibition, AMES Toxicity, Carcinogens, Fish Toxicity, Tetrahymena Pyriformis Toxicity, Honey Bee Toxicity, Biodegradation, Acute Oral Toxicity and Carcinogenicity (Three-class) were considered.

**Table 8:** Pharmacokinetic analysis for compound 2.

2.0.0.1. ADMET Predicted Profile — Classification		
Model	Result	Probability
**Absorption**		
Blood-Brain Barrier	BBB-	0.7736
Human Intestinal Absorption	HIA+	0.9654
Caco-2 Permeability	Caco2-	0.7115
P-glycoprotein Substrate	Substrate	0.8403
P-glycoprotein Inhibitor	Inhibitor	0.5901
	Non-inhibitor	0.7557
Renal Organic Cation Transporter	Non-inhibitor	0.7975
**Distribution**		
Subcellular localization	Mitochondria	0.7917
**Metabolism**		
CYP450 2C9 Substrate	Non-substrate	0.8451
CYP450 2D6 Substrate	Non-substrate	0.7247
CYP450 3A4 Substrate	Substrate	0.5556
CYP450 1A2 Inhibitor	Non-inhibitor	0.8795
CYP450 2C9 Inhibitor	Non-inhibitor	0.9156
CYP450 2D6 Inhibitor	Non-inhibitor	0.8884
CYP450 2C19 Inhibitor	Non-inhibitor	0.8389
CYP450 3A4 Inhibitor	Inhibitor	0.7773
CYP Inhibitory Promiscuity	Low CYP Inhibitory Promiscuity	0.7518
**Excretion**		
**Toxicity**		
Human Ether-a-go-go-Related Gene Inhibition	Weak inhibitor	0.9334
	Inhibitor	0.5000
AMES Toxicity	Non-AMES toxic	0.8512
Carcinogens	Non-carcinogens	0.8949
Fish Toxicity	High FHMT	0.9440
Tetrahymena Pyriformis Toxicity	High TPT	0.9826
Honey Bee Toxicity	Low HBT	0.7234
Biodegradation	Not ready biodegradable	1.0000
Acute Oral Toxicity	III	0.7058
Carcinogenicity (Three-class)	Non-required	0.6860
2.0.0.2. ADMET Predicted Profile — Regression		
**Model**	**Value**	**Unit**
**Absorption**		
Aqueous solubility	−3.2217	LogS
Caco-2 Permeability	0.5699	LogPapp, cm/s
**Toxicity**		
Rat Acute Toxicity	2.4013	LD50, mol/kg
Fish Toxicity	1.4958	pLC50, mg/L
Tetrahymena Pyriformis Toxicity	0.1846	pIGC50, ug/L

**Table 9:** Pharmacokinetic analysis for compound reference compound.

2.0.0.3. ADMET Predicted Profile — Classification		
Model	Result	Probability
**Absorption**		
Blood-Brain Barrier	BBB-	0.9865
Human Intestinal Absorption	HIA-	0.7334
Caco-2 Permeability	Caco2-	0.7487
P-glycoprotein Substrate	Substrate	0.6185
P-glycoprotein Inhibitor	Inhibitor	0.5371
	Non-inhibitor	0.8246
Renal Organic Cation Transporter	Non-inhibitor	0.8988
**Distribution**		
Subcellular localization	Mitochondria	0.4966
**Metabolism**		
CYP450 2C9 Substrate	Non-substrate	0.7494
CYP450 2D6 Substrate	Non-substrate	0.8589
CYP450 3A4 Substrate	Non-substrate	0.5655
CYP450 1A2 Inhibitor	Non-inhibitor	0.9049
CYP450 2C9 Inhibitor	Non-inhibitor	0.9048
CYP450 2D6 Inhibitor	Non-inhibitor	0.9034
CYP450 2C19 Inhibitor	Non-inhibitor	0.8918
CYP450 3A4 Inhibitor	Non-inhibitor	0.9876
CYP Inhibitory Promiscuity	Low CYP Inhibitory Promiscuity	0.8558
**Excretion**		
**Toxicity**		
Human Ether-a-go-go-Related Gene Inhibition	Weak inhibitor	0.9285
	Non-inhibitor	0.7983
AMES Toxicity	Non-AMES toxic	0.8371
Carcinogens	Non-carcinogens	0.9688
Fish Toxicity	High FHMT	0.5760
Tetrahymena Pyriformis Toxicity	High TPT	0.9129
Honey Bee Toxicity	High HBT	0.5435
Biodegradation	Ready biodegradable	0.5467
Acute Oral Toxicity	IV	0.6204
Carcinogenicity (Three-class)	Non-required	0.6721
2.0.0.4. ADMET Predicted Profile — Regression		
**Model**	**Value**	**Unit**
**Absorption**		
Aqueous solubility	−1.3719	LogS
Caco-2 Permeability	−0.4941	LogPapp, cm/s
**Distribution**		
**Metabolism**		
**Excretion**		
**Toxicity**		
Rat Acute Toxicity	1.4609	LD50, mol/kg
Fish Toxicity	1.4865	pLC50, mg/L
Tetrahymena Pyriformis Toxicity	0.1071	pIGC50, ug/L

Moreover, the factors considered for ADMET predicted profile — regression were Aqueous solubility, Caco-2 Permeability (absorption) and Rat Acute Toxicity, Fish Toxicity and Tetrahymena Pyriformis Toxicity (toxicity).

## 4 Experimental Design, Materials and Methods

The investigated cyclic peptide-based compounds were modelled using Spartan ’14 software [[1], [2], [3]] by linking the appropriate atoms to each other via a bond creation tool. The compounds under investigation were optimized using the density functional theory method via 6-31G** as basis set and the electronic descriptors obtained were reported accordingly. In this work, the human insulin-degrading enzyme (PDB id: 2g56) [4] was retrieved from a protein data bank and it was treated and prepared for docking using the induced fit method via molecular operating environment (MOE) software [5,6]. The calculated binding affinity for individual cyclic peptides was acquired and presented.

## Limitations

Cyclic peptides are compounds with high specificity and stability and are expected to play a crucial role in drug discovery. Thus, the advanced method to further probe the activity of cyclic tetra peptide is advisable.

## Ethics Statement

This study does not involve studies with animals and humans.

## CRediT authorship contribution statement

**Abel K. Oyebamiji:** Conceptualization, Methodology, Data curation, Writing – original draft, Visualization, Investigation, Writing – review & editing. **Faith Eniola Olujinmi:** Conceptualization, Methodology, Data curation, Writing – original draft, Visualization, Investigation, Writing – review & editing. **Halleluyah O. Aworinde:** Data curation, Visualization, Investigation. **David G. Oke:** Data curation, Visualization, Investigation. **Sunday Adewale Akintelu:** Conceptualization, Methodology, Data curation, Writing – original draft, Visualization, Investigation, Writing – review & editing. **Emmanuel T. Akintayo:** Data curation, Writing – review & editing. **C.O. Akintayo:** Methodology, Data curation, Writing – original draft. **Jonathan O. Babalola:** Methodology, Data curation, Writing – review & editing.

## Data Availability

Dataset on anti-Human Insulin-Degrading Enzyme activities of Cyclic Tetra Peptides: Insight from Insilico Approach (Original data) (Mendeley Data).
